# Terminally ill patients’ and their relatives’ experiences and behaviors regarding complementary and alternative medicine utilization in hospice palliative inpatient care units: a cross-sectional, multicenter survey

**DOI:** 10.1186/s12906-023-03859-3

**Published:** 2023-02-02

**Authors:** Yu-Jia Lin, Hsiao-Ting Chang, Ming-Hwai Lin, Ru-Yih Chen, Ping-Jen Chen, Wen-Yuan Lin, Jyh-Gang Hsieh, Ying-Wei Wang, Chung-Chieh Hu, Yi-Sheng Liou, Tai-Yuan Chiu, Chun-Yi Tu, Bo-Ren Cheng, Tzeng-Ji Chen, Fang-Pey Chen, Shinn-Jang Hwang

**Affiliations:** 1 Department of Family Medicine, Taipei Veterans General Hospital, Taipei, Taiwan; 2 School of Medicine, National Yang Ming Chiao Tung University, Taipei, Taiwan; 3 School of Medicine, National Defense Medical Center, Taipei, Taiwan; 4 Department of Family Medicine, Kaohsiung Veterans General Hospital, Kaohsiung, Taiwan; 5 Kaohsiung Medical University Chung-Ho Memorial Hospital, Kaohsiung, Taiwan; 6 Department of Family Medicine, China Medical University Hospital, Taichung, Taiwan; 7 Department of Family Medicine, Hualien Tzu Chi Hospital, Hualien, Taiwan; 8 Department of Family Medicine, Taichung Veterans General Hospital, Taichung, Taiwan; 9 Department of Family Medicine, National Taiwan University Hospital, Taipei, Taiwan; 10 Department of Family Medicine, Taipei Veterans General Hospital, Taoyuan Branch, Taoyuan, Taiwan; 11 Center for Traditional Medicine, Taipei Veterans General Hospital, Taipei, Taiwan; 12 Institute of Traditional Medicine, National Yang Ming Chiao Tung University, Taipei, Taiwan; 13 En Chu Kong Hospital, New Taipei City, Taiwan

**Keywords:** Behavior, Communication, Complementary medicine, Alternative medicine, Experience, Palliative care, Terminally ill

## Abstract

**Background:**

Terminally ill patients often experience exacerbations of diseases that render mainstream medicine ineffective in relieving symptoms, prompting attempts at complementary and alternative medicine (CAM). This study collected data from terminally ill patients and their relatives to determine differences between CAM use, behavioral patterns, and perceptions of health information about CAM.

**Methods:**

A cross-sectional design using a self-administered questionnaire was adopted. Eight medical institutions in Taiwan with inpatient hospice palliative care units were chosen. Ninety-two terminally ill patients and 267 relatives met the inclusion criteria. The questions concerned the experience of CAM use, the kinds of products/services CAM provided, the purpose of CAM use, the source of CAM information, and the perceptions and attitudes toward CAM.

**Results:**

Both terminally ill patients and their relatives have a high proportion of lifetime and one-year prevalence of CAM use (88.0% vs. 88.4%; *p* = 0.929). CAM use for musculoskeletal and neurological discomfort is higher among terminally ill patients than among their relatives. Relatives/friends are the most frequent sources of information on CAM (53.3% vs. 62.2%; *p* = 0.133). The percentage of terminally ill patients who discontinued mainstream medical treatment because of CAM use was higher than that of their relatives (18.5% vs. 9.3%; *p* = 0.026). More than half the terminally ill patients and their relatives had never been asked about CAM by medical staff (64.1% vs. 66.7%), nor had they informed medical professionals about the use of CAM products and services (63% vs. 66.9%). Random inquiries by medical professionals may be associated with increased disclosure of CAM use (terminally ill patients: odds ratio, 9.75; 95% confidence interval, 1.97–48.35 vs. relatives: odds ratio, 5.61; 95% confidence interval, 2.66–11.83).

**Conclusions:**

The high prevalence and concealment of CAM use in terminally ill patients should be considered. Medical professionals should establish a friendly and barrier-free communication model, encourage patients to share CAM experiences, and provide evidence-based information on the use of CAM products and services, to reduce the potential damage caused by harmful use.

## Background

Near the end of life, terminally ill patients often feel that their physical and mental health is deteriorating, and they suffer from pain, fatigue, nausea and vomiting, anxiety, insomnia, and other symptoms [1, 2]. These discomforts caused by disease progression or treatment-related side effects often place a significant physical and psychological burden on terminally ill patients and severely impact their quality of life [3]. Therefore, how the suffering of terminally ill patients can be alleviated and their final journey of life be made smooth is not only a topic of discussion among medical practitioners but also a goal pursued by patients and their families [4]. Among the many medical options available, complementary and alternative medicine (CAM) is one that can be adopted by terminally ill patients. Whether from the desire to relieve their discomfort, improve both physical and mental health, or try to take control of their lives, many terminally ill patients try to use CAM products or therapies [5, 6].

In recent years, there has been increasing evidence that CAM is effective in the treatment of many specific symptoms or in certain clinical scenarios, and there has been progress in terms of CAM in the field of palliative medicine [4, 7–10]. Towler et al. [11] systematically appraised 17 reviews about acupuncture in supportive and palliative care for cancer, and discovered that acupuncture is beneficial for the treatment of many cancer-related symptoms, including fatigue, nausea, vomiting, pain, dyspnea, hot flushes, dry mouth, and anxiety. Armstrong et al. [12] identified five qualitative studies of people with advanced disease and concluded that aromatherapy, reflexology, and/or massage positively affected the relief of worry and anxiety caused by the disease, as well as well-being in terminally ill patients. In another randomized controlled trial on mindfulness meditation, mindfulness-based stress reduction (MBSR) was deemed effective for the treatment of patients with non-specific chronic pain [13]. These findings provide a rationale for the clinical use of CAM in the field of palliative medicine.

However, improper usage and habits pose risks such us CAM-related adverse effects or delayed diagnosis/treatment [14]. Especially in modern society, where information is rapidly and widely disseminated, CAM users may be exposed to biased or misleading information [15–18]. In addition, because of the authority of mainstream medicine, some CAM users may be worried about how their physicians will react if they disclose their use of CAM [19]. Therefore, if the physician does not initiate a discussion about a patient’s use of CAM, the patient–physician communication may be obstructed, impairing decision-making regarding a patient’s condition [20]. In an analysis of data obtained from a questionnaire among Australian adults, 30.4% (*n* = 221) of participants did not disclose or only partially disclosed CAM use to their physician [21]. Hence, although CAM is receiving increased recognition for its benefits, many obstacles remain to its use in clinical practice.

Considering that CAM usage and related behavioral patterns among terminally ill patients may differ from those of other populations, it is crucial that these be assessed to gauge the potential impact of CAM on this population. The purpose of our research was to investigate the conditions for CAM use, the differences in experiences of CAM use between patients and their relatives, and factors associated with patients and their relatives disclosing their CAM use to medical personnel in hospice and palliative care inpatient facilities in Taiwan.

## Methods

### Study design

This was a descriptive, cross-sectional, non-experimental study involving patients and their relatives in hospice palliative inpatient care units in eight hospitals in Taiwan.

### Ethical consideration

This study was approved by the institutional review boards of all eight hospitals to ensure the protection of research participants.

### Settings and participants

To maximize the representativeness of the research results, we selected hospitals located in the northern, central, southern, and eastern regions of Taiwan. The following hospitals participated: Taipei Veterans General Hospital; Taipei Veterans General Hospital, Taoyuan Branch; National Taiwan University Hospital; Taichung Veterans General Hospital; China Medical University Hospital; Kaohsiung Veterans General Hospital; Chi Mei Hospital; and Hualien Tzu Chi Hospital. We enrolled patients and their relatives at the hospice palliative care inpatient units in the above eight hospitals from October 2015 to July 2016. In addition to terminal cancer, patients with the following non-cancer terminal diseases were also eligible for hospice palliative inpatient care: (1) motor neuron disease; (2) senile and presenile organic psychotic conditions; (3) other brain diseases; (4) heart failure; (5) chronic airway obstruction, not elsewhere classified; (6) other diseases of the lung; (7) chronic liver disease and cirrhosis; (8) acute renal failure; and (9) chronic renal failure and end stage renal disease. All the patients enrolled in this study meet the definition of “terminal patients” in Article 3 of Taiwan's Hospice Palliative Care Act: “terminal patients refers to those who suffer from serious injury or illness, and are diagnosed by physicians as incurable, and there is medical evidence that the prognosis is poor within the near future.” Each patient had at least one relative who was also enrolled in this study together with the patient. Eligibility for relatives is based on the provisions of Taiwan's civil code, which include mainly the following three categories: (1) spouses, (2) blood relatives, and (3) relatives by marriage. The exclusion criteria were patients and relatives who were (1) under 20 years of age, (2) those who were unable to respond to the questionnaire, and (3) those who refused to answer the questionnaire.

### Outcome measures and data collection procedures

We designed this study based on the clinical and research experience of the authors, all of whom are professionals in the fields of palliative medicine and CAM. To fulfill the aims of this study, we developed a questionnaire. A preliminary questionnaire was established following comprehensive literature reviews; thereafter, experts in related fields were invited for its validation. We conducted a pilot test involving 20 patients and their relatives to test the reliability of the questionnaire; we adjusted its contents according to the results of the pilot test.

After optimization and correction, the final version of the questionnaire consisted of two parts. The first part was designed to assess the demographic characteristics of the participants, including their gender, age (< 45, 45–64, or ≥ 65 years), marital status (never married, married, widowed, or divorced), educational level (< 9, 9–12, or ≥ 13 years), religiosity (yes or no), area of residence (northern, central, southern, or eastern region of Taiwan), and annual household income (≤ NT$239,999, NT$240,000–NT$479,999, NT$480,000–NT$719,999, or ≥ NT$720,000 [New Taiwan dollar]).

The second part of the questionnaire comprised five major components. The first evaluated participants’ experiences with CAM use, with the following four questions: (1) Have you ever used CAM? (2) Have you used CAM in the past year? (3) How often have you utilized CAM in the past year? (4) Have you ever discontinued mainstream medicine because of CAM use? The second component investigated the distribution of use of common CAM modalities; the participants selected from a list which CAM types they had ever used. These included traditional Chinese herbal medicine (prescribed by a traditional Chinese medicine practitioner), herbal medicine (self-use), concentrated Chinese medicine granules, health foods, vitamins/dietary supplements, chiropractic/osteopathy/subluxation reduction, tuina/massage, acupressure, acupuncture, aromatherapy, energy healing, mind–body interventions, exercise, and folk remedies. Chiropractic/osteopathy/subluxation reduction all belong to manipulative therapy: a type of physical therapy that uses hand movements to improve mobility in joints, soft tissues, and skeletal muscles. The third component used a multiple-choice list examining which affected biological systems the participants wanted to improve via CAM, including the musculoskeletal, nervous, cardiovascular, respiratory, gastrointestinal, urinary, and endocrine systems. The fourth component used a multiple-choice list investigating sources from which participants obtained information about CAM, including medical practitioners, relatives/friends, other patients, TV/radio, the Internet, books/newspapers/magazines, and lectures/associations. The fifth component contained the following questions to assess the present condition of CAM-related communication between participants and healthcare providers: (1) Has a medical practitioner ever asked you whether you were currently using CAM? (2) Have you ever informed a medical practitioner that you were using CAM? If the answer to the second question was “No,” the participants had to answer the next question: (3) What are your main reasons for not informing your medical practitioner of your use of CAM?

### Statistical analysis

All data were analyzed using PASW Statistics for Windows (Version 18.0, SPSS Inc., Chicago, IL, USA). Cohen's Kappa was used to measure the reliability of the questionnaire. Chi-square tests were used to analyze differences in categorical demographic characteristics, including gender, age, marital status, educational level, religiosity, area of residence, and annual household income between terminally ill patients and their relatives. Differences in the two groups’ experiences with CAM use, the distribution of utilization of CAM modalities, the therapeutic purpose of CAM, and their sources of information of CAM were also assessed using chi-square tests. We used binary logistic regression models to explore the factors in each group associated with informing medical practitioners about CAM use. The following independent variables were entered into the models: gender, age, marital status, educational level, religiosity, area of residence, annual household income, CAM use in the past year, discontinuation of mainstream medicine, and having been asked about CAM utilization by medical practitioners. Odds ratios (ORs), 95% confidence intervals (CIs), and *p* values were presented for each of these variables. For all comparisons, two-tailed tests were used, and statistical significance was defined as *p* < 0.05.

## Results

### Demographic characteristics of participants

Among the 359 participants who completed the questionnaires (response rate: 82.7%), 92 were terminally ill patients and 267 were their relatives, with 434 questionnaires distributed in total. The demographic characteristics that differed between patients and their relatives were gender (*p* = 0.033), age (*p* < 0.0001), marital status (*p* < 0.0001), educational level (*p* < 0.0001), and area of residence (*p* < 0.0001). There were no statistical differences in religiosity or annual household income. Table 1 presents the detailed demographic characteristics of the participants.

**Table 1:** Characteristics of the participants

Characteristics	Patients, n (%)	Relatives, n (%)	*p* value (χ^2^ test)
Total	92 (25.6)	267 (74.4)	
Gender			0.033
Male	35 (38.0)	136 (50.9)	
Female	57 (62.0)	131 (49.1)	
Age (years)^a^			< .0001
< 45	24 (26.1)	19 (7.1)	
45–64	44 (47.8)	78 (29.2)	
≥ 65	23 (25)	167 (62.5)	
Missing	1 (1.1)	3 (1.1)	
Marital status^a^			< .0001
Single/Never married	20 (21.7)	16 (6.0)	
Married	60 (65.2)	181 (67.8)	
Widowed/Divorced	12 (13.0)	68 (25.5)	
Missing	0 (0)	2 (0.75)	
Educational level^a^			< .0001
< 9 years	22 (23.9)	134 (50.2)	
9–12 years	25 (27.2)	62 (23.2)	
≥ 13 years	45 (48.9)	67 (25.1)	
Missing	0 (0)	4 (1.5)	
Religion^a^			0.196
No	17 (18.5)	34 (12.7)	
Yes	75 (81.5)	228 (85.4)	
Missing	0 (0)	5 (1.9)	
Region in Taiwan^a^			< .0001
Northern	34 (37.0)	174 (65.2)	
Central	9 (9.8)	42 (15.7)	
Southern	14 (15.2)	37 (13.9)	
Eastern	35 (38.0)	13 (4.9)	
Missing	0 (0)	1 (0.4)	
Annual household income^a,b^			0.265
≤ $239,999	24 (26.1)	52 (19.5)	
$240,000–$479,999	26 (28.3)	63 (23.6)	
$480,000–$719,999	20 (21.7)	54 (20.2)	
≥ $720,000	18 (19.6)	76 (28.5)	
Missing	4 (4.3)	22 (8.2)	

^a^Answers that could not be classified (containing unknown or unclear) were excluded

^b^New Taiwan Dollar, NTD

### CAM use

As indicated in Table 2, the frequencies with which patients and their relatives used CAM during the past year differed statistically significantly (*p* = 0.035). Further, compared with their relatives, a significantly higher ratio of terminally ill patients had ever discontinued mainstream medicine because of CAM use (patients: 18.5%; relatives: 9.3%; *p* = 0.026). Regarding the prevalence of CAM use in a lifelong and 12-month period, there were no significant differences between patients and their relatives.

**Table 2:** Differences in experience in CAM use between patients and their relatives

	Patients, n (%)	Relatives, n (%)	*p* value (χ^2^ test)
Had you ever used CAM in your life?			0.929
Yes	81 (88.0)	236 (88.4)	
No	11 (12.0)	31 (11.6)	
Experience of CAM use in the past year^a^			
Yes	68 (73.9)	196 (73.4)	0.928
No	23 (25)	68 (25.5)	
Missing	1 (1.1)	3 (1.1)	
Frequency of CAM use in the past year^a^			0.035
Never	23 (25)	68 (25.5)	
Once	6 (6.5)	19 (7.1)	
Sometimes	41 (44.6)	79 (29.6)	
Often	21 (22.8)	98 (36.7)	
Missing	1 (1.1)	3 (1.1)	
Had you ever discontinued mainstream medicine because of CAM use?			0.026
Yes	15 (18.5)	22 (9.3)	
No	66 (81.5)	214 (90.7)	

^a^Answers that could not be classified (containing unknown or unclear) were excluded

### Distribution of CAM utilization

**Fig. 1:**
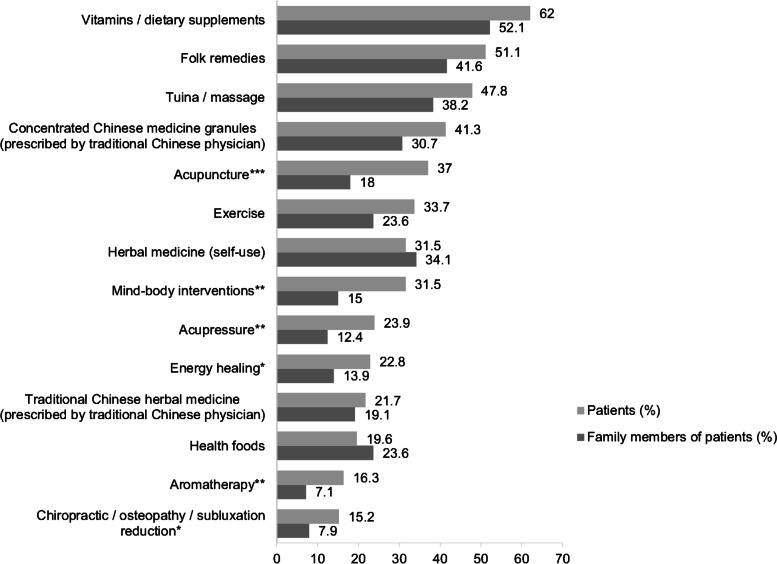
Distribution of CAM utilization by terminally ill patients and their relatives (Multiple answers possible) (Patients, *n*=92; Relatives, *n*=267). *Note: Significant at 0.05 level with Chi-square test. **Note: Significant at 0.01 level with Chi-square test. ***Note: Significant at 0.001 level with Chi-square test. CAM: complementary and alternative medicine

Figure 1 displays the distribution of use of different CAM modalities among terminally ill patients (*n* = 92) and their relatives (*n* = 267). We discovered that the top three most utilized CAM modalities were the same for patients and their relatives, namely vitamins/dietary supplements (62.0% vs. 52.1%, respectively), folk remedies (51.1% vs. 41.6%, respectively), and tuina/ massage (47.8% vs. 38.2%, respectively). Compared with their relatives, terminally ill patients had significantly higher rates of use of acupuncture (18.0% vs. 37.0%, respectively; *p* < 0.0001), mind–body interventions (15.0% vs. 31.5%, respectively; *p* = 0.001), acupressure (12.4% vs. 23.9%, respectively; *p* = 0.008), energy healing (13.9% vs. 22.8%, respectively; *p* = 0.044), aromatherapy (7.1% vs. 16.3%, respectively; *p* = 0.009), and chiropractic/osteopathy/subluxation reduction (7.9% vs. 15.2%, respectively; *p* = 0.040).

### Affected biological system leading to CAM use

**Fig. 2:**
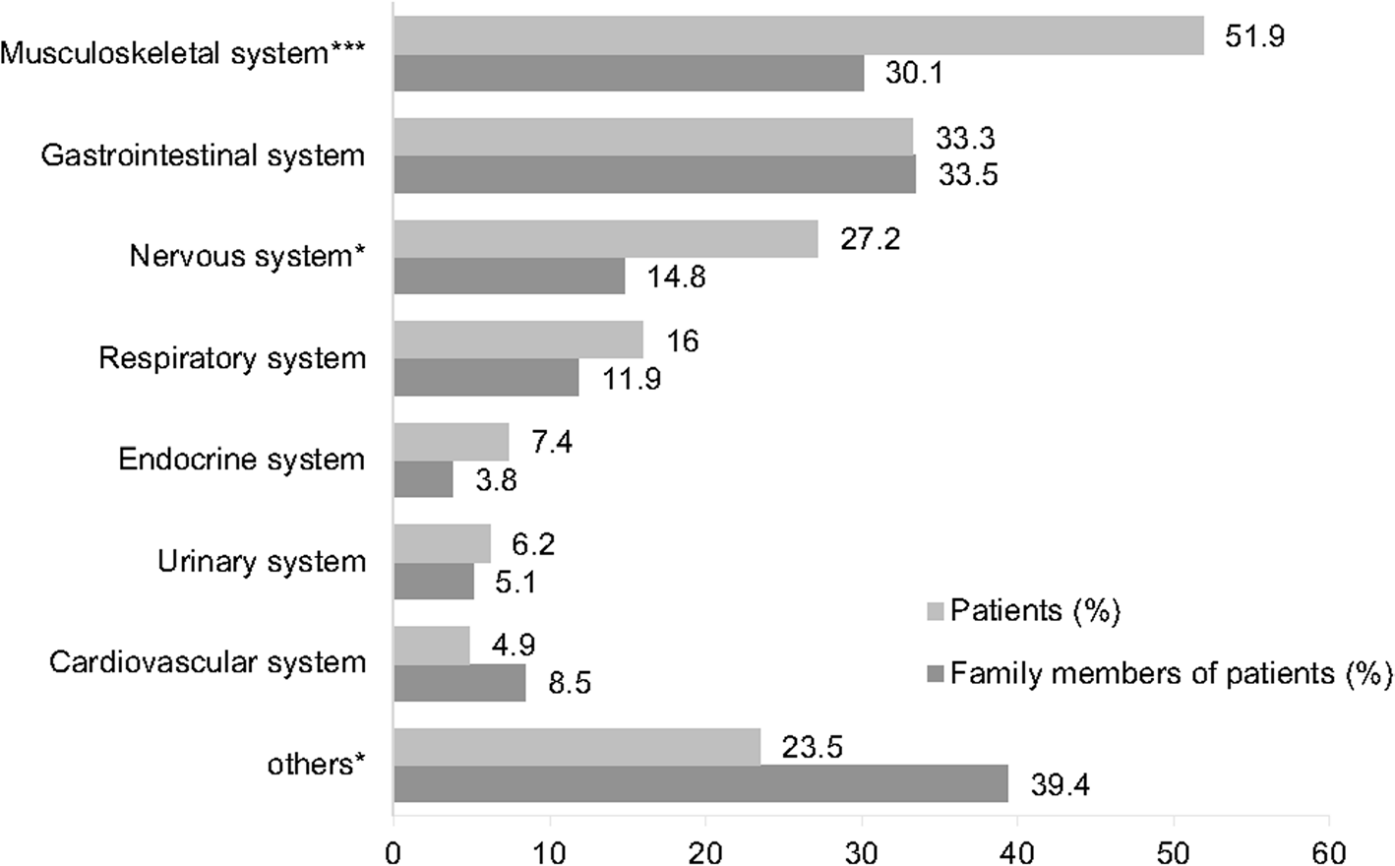
The personal expectation of CAM use for improving the discomfort of which biological system (Multiple answers possible) (Patients, *n*=81; Relatives, *n*=236). *Note: Significant at 0.05 level with Chi-square test. ***Note: Significant at 0.001 level with Chi-square test. CAM: complementary and alternative medicine

Figure 2 demonstrates the different biological systems for which patients and their relatives had sought relief through CAM (patients: *n* = 81 and relatives: *n* = 236). The biological systems for which patients and their relatives most commonly sought relief were the musculoskeletal (51.9% vs. 30.1%), gastrointestinal (33.3% vs. 33.5%), and nervous (27.2% vs. 14.8%) systems. Compared with relatives, a higher proportion of patients used CAM products and/or services for symptomatic relief of the musculoskeletal and nervous systems (musculoskeletal system: *p* =  < 0.0001 and nervous system: *p* = 0.013). Additionally, a higher proportion of relatives than patients used CAM for “other” purposes, namely for diseases not associated with a specific biological system or which could not be classified into any biological system.

### Sources of information about CAM

The introduction to or recommendation of CAM by relatives or friends was the most common source of information about CAM, both for patients and their relatives (53.3% vs. 62.2%, respectively; *p* = 0.133), followed by suggestions by medical practitioners (28.3% vs. 22.5%, respectively; *p* = 0.262). Although the amount of information about CAM obtained through lectures or associations was relatively low compared with other sources, more patients obtained information in this way than did their relatives (10.9% vs. 4.5%, respectively; *p* = 0.028).

### Disclosure of CAM use to medical practitioners

**Fig. 3:**
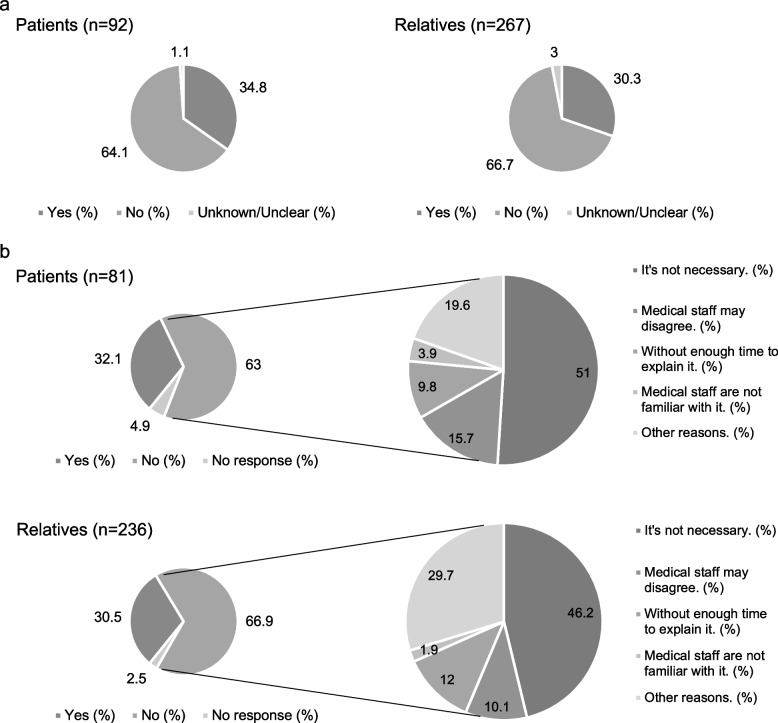
Disclosure of CAM use to medical practitioners a. The experience of having been asked about CAM use by medical staff b. The status of disclosure of CAM products or services to medical personnel. Reported the answers to the following questions: (1) Do you inform medical staff about CAM use? (Left circle graph) (2) What is the main reason for not informing medical staff about CAM use? (Right circle graph). CAM: complementary and alternative medicine

Figure 3 depicts the communication regarding CAM use between participants and medical practitioners. As illustrated in Fig. 3a, about two-thirds of the participants indicated that medical practitioners had never asked them whether they were making use of CAM products and/or services (patients: 64.1%; relatives: 66.7%). Similarly, Fig. 3b illustrates that approximately two-thirds of the participants had not informed their medical practitioners about their use of CAM (patients: 63.0%; relatives: 66.9%). The most common reason for not disclosing CAM use to medical practitioners was “It’s not necessary,’’ for both patients (51.0%) and their relatives (46.2%).

After excluding patients and their relatives who did not answer all the questions, 77 patients and 230 relatives were included in the binary logistic regression model to explore the factors related to the disclosure of CAM use to medical practitioners. As indicated in Table 3, concerning the patients’ relatives, men were significantly less likely than women to disclose CAM use (OR: 0.47; 95% CI: 0.22–0.98); those aged 45–64 and ≥ 65 years were significantly more likely to disclose CAM use than were those who were < 45 years old (OR: 8.76; 95% CI: 1.48–51.79 and OR: 8.49; 95% CI: 1.30–55.28, respectively); and those who had often used CAM in the past year were significantly more likely to disclose their use of CAM than those who had not (OR: 2.01; 95% CI: 1.01–4.00). In addition, both the patients and their relatives were more willing to disclose their use of CAM if medical practitioners enquired about it (OR: 9.75, 95% CI: 1.97– 48.35 and OR: 5.61, 95% CI: 2.66–11.83, respectively).

**Table 3:** Factors related to the disclosure of CAM use to medical personnel (Patients, *n* = 77; Relatives, *n* = 230)

	Patients			Relatives		
Variable	Odds Ratio	95% CI^a^	*p* value	Odds Ratio	95% CI^a^	*p* value
Gender						
Female^b^	Reference			Reference		
Male	0.56	0.10–3.14	0.511	0.47	0.22–0.98	0.044
Age (years)						
< 45^b^	Reference			Reference		
45–64	3.21	0.42–24.78	0.263	8.76	1.48–51.79	0.017
≥ 65	7.51	0.82–68.49	0.074	8.49	1.30–55.28	0.025
Marital status						
Married^b^	Reference			Reference		
Single/Never married	3.28	0.36–30.01	0.293	5.04	0.99–25.80	0.052
Widowed/Divorced	0.26	0.03–2.63	0.253	0.87	0.38–2.00	0.749
Educational level						
< 9 years^b^	Reference			Reference		
9–12 years	0.45	0.05–4.13	0.483	1.10	0.43–2.79	0.846
≥ 13 years	1.00	0.13–7.66	0.999	2.12	0.86–5.21	0.102
Religion						
No^b^	Reference			Reference		
Yes	1.31	0.18–9.43	0.787	1.23	0.36–4.26	0.739
Region in Taiwan						
Central, Southern, or Eastern^b^	Reference			Reference		
Northern	0.19	0.03–1.09	0.062	1.04	0.48–2.24	0.925
Annual household income^c^						
≤ $239999^b^	Reference			Reference		
$240,000–$479,999	1.02	0.14–7.58	0.982	0.90	0.31–2.62	0.851
$480,000–$719,999	0.32	0.02–4.92	0.416	0.69	0.23–2.12	0.522
≥ $720,000	0.43	0.05–3.51	0.432	0.83	0.29–2.39	0.730
Frequent use of CAM in the past year						
No^b^	Reference			Reference		
Yes	1.32	0.22–8.10	0.762	2.01	1.01–4.00	0.048
Ever discontinued mainstream medicine use						
No^b^	Reference			Reference		
Yes	0.56	0.09–3.51	0.533	2.44	0.82–7.28	0.110
Experience of having been asked about CAM use by medical staff						
No^b^	Reference			Reference		
Yes	9.75	1.97–48.35	0.005	5.61	2.66–11.83	< 0.001

^a^CI: confidence interval

^b^Reference categories

^c^New Taiwan Dollar, NTD

## Discussion

In this study involving terminally ill patients and their relatives in eight hospice palliative care inpatient units, a comprehensive analysis of CAM use was undertaken via a survey. There were several statistically significant findings in terms of use, behavior, and attitude regarding CAM use. First, our results demonstrated a high frequency of CAM use and a diversity of CAM products and services used among the participants. Vitamins/dietary supplements are the most used CAM items by both terminally ill patients and their relatives. Further analysis revealed that terminally ill patients had a higher utilization rate of acupuncture, acupressure, physical and mental therapy, energy therapy, aromatherapy, and manipulative therapy than their relatives had. Second, our results revealed that relatives and friends were the main source of information about CAM for both terminally ill patients and their relatives. A statistically significantly higher proportion of terminally ill patients had stopped mainstream medicine before because of CAM use compared with their relatives. Third, we discovered that most of the participants did not provide their medical practitioners with information about CAM use. Moreover, our results suggest that medical practitioners can assist participants in disclosing their CAM use by initiating such a conversation.

The results of this survey showed that terminally ill patients and their relatives have different goals for relieving physical symptoms. Compared with their relatives, a higher proportion of terminally ill patients use acupuncture, acupressure, energy healing, and chiropractic/osteopathic/subluxation reduction for musculoskeletal and neurological symptoms. As the disease progresses or adverse effects occur during treatment, symptoms related to the musculoskeletal system and the nervous system often cause pain and discomfort to the patient and significantly impact an individual's ability to participate in daily activities and their quality of life. According to a review of the literature on cancer-related pain, 76% of terminally ill patients experience pain, about half of which are nociceptive, and about one-third experience neuropathic pain alone or in combination with nociceptive pain [22]. Apparently, effective pain control remains a challenge for clinical practitioners.

Today, some CAM products or services have a certain level of evidence for cancer pain control. A meta-analysis of 14 randomized controlled trials with a total of 920 patients suggested that acupuncture and acupressure were associated with reduced analgesic use and cancer pain [23]. Regarding the efficacy of energy healing as cancer pain control, a randomized clinical trial of 24 patients with cancer pain showed that Reiki therapy as an adjunct to standard opioid therapy for cancer pain could better improve subjective pain and quality of life [24]. In another randomized clinical trial evaluating the efficacy of therapeutic touch, a total of 90 male cancer pain patients were randomly assigned to one of three groups (intervention, control, and placebo group), and it was found that the patients in the intervention group, who received therapeutic touch, experienced significantly better pain control than the other two groups [25]. Manipulative therapy is widely used to treat musculoskeletal disorders and low back and neck pain, and has been documented to be effective in nociceptive/musculoskeletal pain [26–28]. For pain management in cancer patients, one review summarizes the current evidence suggesting that manipulative therapy is also a valid intervention for cancer-related pain, with immediate treatment effects [29].

However, the application of manipulative therapy to cancer patients may have potential risks, especially in patients with bone metastases, which may increase the chance of pathological fractures. Therefore, malignancies, including malignant bone tumors, have been identified as an absolute contraindication to manipulation therapy [30]. When considering treatment options for CAM in clinical practice, we need to assess not only the existence of evidence for an indication for treatment but also the strength of the evidence, the certainty of effect, and the risk and burden to the patient. The benefits of the treatment should be weighed against the potential risks and whether the treatment is in the best interests of the terminally ill patient. Therefore, appropriate integration of evidence-based CAM into comprehensive pain management plans for palliative care and incorporation of CAM into clinical practice guidelines can not only optimize pain control in terminally ill patients but also provide healthcare professionals with a foundation for the safe use of CAM.

Interestingly, medical personnel, as the main providers of medical professional information, are not the primary source of CAM information neither for terminally ill patients nor for their relatives. In the CAM discipline, where authoritative information resources are scarce, anecdotal evidence or social proof often becomes an important factor in patients’ intention to use [31]. Studies have shown that cancer patients, especially men, pay special attention to the advice and opinions of “trustworthy people” when choosing CAM products or services, especially female family members, who played an important role in guiding patients to use CAM [32]. In addition, our survey found that traditional information media such as books, newspapers, magazines, TV channels, and radio stations seem to be more popular with terminally ill patients and their relatives than the Internet. Although the Internet provides a wealth of useful information about CAM, many users say that the most difficult part of searching for health information on the Internet is how to use more accurate search terms. Clutter, irrelevant information, and pop-ups may also impede the search for health information, all of which can degrade a patient’s experience with the Internet and influence usage intent [33]. Although there are many channels for obtaining CAM information, medical personnel are still an important source of CAM information. When cancer patients choose to use CAM, the advice of oncology health professionals is often an important reference in the decision-making process [34]. Therefore, how to improve the cultivation of CAM professional knowledge and create an open and friendly communication environment between medical staff and patients so that patients can learn accurate information about CAM, is an important issue for medical personnel.

A disturbing finding was also uncovered in our investigation. Compared with their relatives, a higher proportion of terminally ill patients who have used CAM had ceased using mainstream medicine because of their CAM use. Rosenstock and Becker have explained that (1) an individual's subjective assessment of the likelihood of developing a disease (perceived susceptibility), (2) an individual's subjective perception of the severity of a disease or not receiving treatment (perceived severity), (3) the benefits of changing behavior (perceived benefits), and (4) difficulties encountered in personal behavior change (perceived barriers) may all be important factors that affect individual cognition and healthy behavior [35]. Among these four variables, perceived susceptibility and perceived severity provide the motivation for action, and the measurement and comparison of perceived benefits and perceived barriers may influence an individual's choice of the best course of action [35]. Additionally, prospect theory attempts to analyze the treatment decision-making patterns of terminally ill patients and assumes that the patients’ health reference point is a key point in determining which treatment option a patient chooses [36]. Although the location of the health reference point is not known from this study, it is clear that there are differences in disease severity and individual treatment experience between terminally ill patients and their families [36]. How the distribution of health reference point locations between terminally ill patients and other populations can be quantified, and exploring differences in treatment decisions, will be an interesting and developing issue.

However, our results indicated that medical practitioners do not seem to play the important role of gatekeepers in the evaluation of CAM use and behavior of patients. More than half of the participants had never been asked by their medical practitioners about their use of CAM. Several perspectives may explain this finding. For example, a survey of physicians in the Denver, Colorado area found that insufficient CAM education rendered it difficult for physicians to discuss CAM with patients [37]. Brief clinical encounter time also limited discussions on CAM use [38]. Moreover, we discovered that two-thirds of terminally ill patients and their relatives did not inform their healthcare providers of their current or previous use of CAM products or services, similar to the results of other studies [39–41]. Further analysis of the reasons for this non-disclosure in this study revealed that the participants believe that it is not necessary to inform their medical practitioners of their CAM use (patients: 51.0%, relatives: 46.2%). Such belief may result from overconfidence in the safety of CAM. For instance, MacLennan et al. [42, 43] revealed that most of the general public assume that CAM products and therapies are safe. CAM treatments touted as "natural" by mass media may also reinforce beliefs that such treatments are hazard-free, increasing the risk of harm to users [44]. Another reason why participants in this study tended to refuse disclosing their CAM use to their medical practitioners was their concern that such use would be met with opposition (patients: 15.7%, relatives: 10.1%). A study of chronically ill Japanese patients also investigated this issue and suggested that fear of refutation and anger from physicians is a possible reason influencing disclosure to physicians [45]. In addition, patients’ anticipation of physicians’ negative perception, may affect the candid communication between physicians and patients [46]. To ensure the medication-related safety of CAM users, medical practitioners have a responsibility to establish trust and an open line of communication with their patients. The implementation of shared decision-making and the introduction of decision-making aids can effectively eliminate the gap between medical staff and terminally ill patients, improve the effectiveness of physician–patient communication and patient health awareness, and create an ideal physician‒patient relationship. Our research points out that both terminally ill patients and their relatives would be more willing to share information related to CAM use (9.8 and 5.6 times more likely, respectively) if their medical practitioners enquire about it. This result is highly consistent with previous reports [26]. By extension, physicians should receive more comprehensive CAM education, and encourage the use of evidence-based patient decision aids to share accurate information and precautions with patients, which provides the patient with all options available for consideration and supports them in making medical decisions consistent with their preferences. Health policy makers should systematically incorporate and enforce policies that promote improved CAM disclosure and comprehensive CAM education for medical professionals in the healthcare institutions’ quality management systems.

The main strength of our study is that data collection was not limited to one place but was distributed across eight medical institutions in the northern, central, southern, and eastern areas of Taiwan. The multicenter nature of our study improves its representativeness. However, this study was also subject to several potential limitations. First, all data were based on self-reported answers to a questionnaire; therefore, a certain degree of recall bias was unavoidable. Second, the survey-based nature of this study posed a risk of selection bias, as individuals who have strong convictions regarding CAM were more likely to complete the questionnaire. Additionally, partial characteristics between terminally ill patients and their relatives were different, which may have affected the comparability of the results. Finally, although variables were carefully selected for inclusion in the regression model, there could have been unknown confounding factors for which we did not control. Future studies should attempt to better comprehend the factors that improve the likelihood that terminally ill patients and their relatives will inform their medical practitioners about their use of CAM products and/or services. In this study, we did not specifically investigate the type of mainstream medicine discontinued because of CAM use and the purpose of CAM use that was substituted after discontinuation of mainstream medicine, although we observed a higher proportion of terminally ill patients discontinuing mainstream medicine; therefore, we could not determine whether it was attributable to specific causes or just incidental findings, which requires a more detailed analysis in future studies.

## Conclusions

This nationwide survey in hospice palliative care inpatient units confirmed the universality and diversity of CAM use among terminally ill patients and their relatives. Our results suggest that terminally ill patients and their relatives often derive information about CAM from sources other than medical professionals and tend to avoid mentioning their use of CAM to their medical practitioners. Additionally, the higher proportion of terminally ill patients, compared with their relatives, who have discontinued mainstream medicine treatment because of their use of CAM, also warrant attention. Healthcare providers should routinely inquire and investigate patients’ past and current use of CAM products and/or services, and provide them with correct information, including about the harms or risks of CAM use. Health policy makers should develop specific policies to improve CAM disclosure and clinical practice training on CAM for healthcare professionals. Establishing effective communication with their patients will increase CAM users’ willingness to share related information with their healthcare providers, thereby reducing the risks and hazards posed by CAM.

## Data Availability

The datasets generated and/or analyzed during the current study are not publicly available because of privacy and ethical concerns but are available from the corresponding author on reasonable request.

## Abbreviations

CAM: Complementary and alternative medicine
CI: Confidence interval
IRB: Institutional review board
MBSR: Mindfulness-based stress reduction
NA: Not applicable
NT$: New Taiwan dollar
OR: Odds ratio
TV: Television
WHO: World Health Organization
